# Use of Mechanical Chest Compression for Resuscitation in Out-Of-Hospital Cardiac Arrest—Device Matters: A Propensity-Score-Based Match Analysis

**DOI:** 10.3390/jcm12134429

**Published:** 2023-06-30

**Authors:** Roberto Primi, Sara Bendotti, Alessia Currao, Giuseppe Maria Sechi, Gianluca Marconi, Greta Pamploni, Gianluca Panni, Davide Sgotti, Ettore Zorzi, Marco Cazzaniga, Umberto Piccolo, Daniele Bussi, Simone Ruggeri, Fabio Facchin, Edoardo Soffiato, Vincenza Ronchi, Enrico Contri, Paola Centineo, Francesca Reali, Luigi Sfolcini, Francesca Romana Gentile, Enrico Baldi, Sara Compagnoni, Federico Quilico, Luca Vicini Scajola, Clara Lopiano, Alessandro Fasolino, Simone Savastano, all the Lombardia CARe Researchers

**Affiliations:** 1 Division of Cardiology, Fondazione IRCCS Policlinico San Matteo, 27100 Pavia, Italyfrancescaromanagentile49@gmail.com (F.R.G.); enrico.baldi@unipv.it (E.B.); sara.compagnoni01@universitadipavia.it (S.C.); federico.quilico01@universitadipavia.it (F.Q.);; 2 Department of Public Health, Experimental and Forensic Medicine, Section of Biostatistics and Clinical Epidemiology, University of Pavia, 27100 Pavia, Italy; 3 Agenzia Regionale dell’Emergenza Urgenza (AREU) Lombardia, 20124 Milan, Italy; g.sechi@areu.lombardia.it (G.M.S.);; 4 AAT Pavia—Agenzia Regionale Emergenza Urgenza (AREU) c/o Fondazione IRCCS Policlinico San Matteo, 27100 Pavia, Italy; 5 AAT Brescia—Agenzia Regionale Emergenza Urgenza (AREU) c/o ASST Degli Spedali Civili di Brescia, 25100 Brescia, Italydavidesgotti@gmail.com (D.S.); 6 AAT Como—Agenzia Regionale Emergenza Urgenza (AREU) c/o ASST Lariana (CO), 22079 Como, Italy; 7 AAT Cremona—Agenzia Regionale Emergenza Urgenza (AREU) c/o ASST di Cremona, 26100 Cremona, Italy; 8 AAT Mantova—Agenzia Regionale Emergenza Urgenza (AREU) c/o ASST di Mantova, 46100 Mantova, Italy; fabio.facchin@gmail.com (F.F.);; 9 AAT Pavia—Agenzia Regionale Emergenza Urgenza (AREU) c/o ASST Pavia, 27100 Pavia, Italy; 10 AAT Varese—Agenzia Regionale Emergenza Urgenza (AREU) c/o ASST dei Sette Laghi, 21100 Varese, Italy; p.centineo@areu.lombardia.it; 11 AAT Lodi—Agenzia Regionale Emergenza Urgenza (AREU) c/o ASST di Lodi, 26900 Lodi, Italy; 12 Department of Molecular Medicine, Section of Cardiology, University of Pavia, 27100 Pavia, Italy

**Keywords:** cardiopulmonary resuscitation, cardiac arrest, resuscitation, survival, mechanical chest compression

## Abstract

Background. Devices for mechanical cardiopulmonary resuscitation (CPR) are recommended when high quality CPR cannot be provided. Different devices are available, but the literature is poor in direct comparison studies. Our aim was to assess whether the type of mechanical chest compressor could affect the probability of return of spontaneous circulation (ROSC) and 30-day survival in Out-of-Hospital Cardiac Arrest (OHCA) patients as compared to manual standard CPR. Methods. We considered all OHCAs that occurred from 1 January 2015 to 31 December 2022 in seven provinces of the Lombardy region equipped with three different types of mechanical compressor: Autopulse^®^(ZOLL Medical, MA), LUCAS^®^ (Stryker, MI), and Easy Pulse^®^ (Schiller, Switzerland). Results. Two groups, 2146 patients each (manual and mechanical CPR), were identified by propensity-score-based random matching. The rates of ROSC (15% vs. 23%, *p* < 0.001) and 30-day survival (6% vs. 14%, *p* < 0.001) were lower in the mechanical CPR group. After correction for confounders, Autopulse^®^ [OR 2.1, 95%CI (1.6–2.8), *p* < 0.001] and LUCAS^®^ [OR 2.5, 95%CI (1.7–3.6), *p* < 0.001] significantly increased the probability of ROSC, and Autopulse^®^ significantly increased the probability of 30-day survival compared to manual CPR [HR 0.9, 95%CI (0.8–0.9), *p* = 0.005]. Conclusion. Mechanical chest compressors could increase the rate of ROSC, especially in case of prolonged resuscitation. The devices were dissimilar, and their different performances could significantly influence patient outcomes. The load-distributing-band device was the only mechanical chest able to favorably affect 30-day survival.

## 1. Introduction

Out-of-Hospital Cardiac Arrest (OHCA) is one of the leading causes of death in industrialized countries [1]. In the European Union, 300,000 to 700,000 cases of OHCA are recorded every year, corresponding to an incidence of 102–207 in every 100,000 inhabitants [2]. The outcomes of OHCA patients are influenced by a series of actions, known as “chain of survival” [3], among which cardiopulmonary resuscitation (CPR) takes on a pivotal role. A wealth of literature on the link between CPR quality and survival has been published [4], and mechanical devices performing chest compressions have been designed and increasingly used in clinical practice to provide patients with high quality CPR. These devices can be clustered into three different categories: piston-driven devices, as LUCAS^®^ (Stryker, MI), conceptually more similar to manual CPR and generally supported by the “cardiac pump” theory; load-distributing-band devices, as Autopulse^®^ (ZOLL Medical, MA), based on the “thoracic pump” theory [5]; and, finally, a combination of piston and band type, such as Easy Pulse^®^ (Schiller, Switzerland).

“Cardiac pump” and “Thoracic pump” are two proven theories explaining how blood flows during CPR6. The former is more similar to the normal cardiac function so that the mitral valve is closed, the aortic valve is open, and blood physiologically flows during chest compressions. The thoracic pump theory is more complex: chest compressions cause a general increase in intrathoracic pressure, which is transmitted to all cardiac chambers and vessels in the thorax. This increased pressure generates an arterio-venous pressure gradient that results in a forward blood flow thanks to the presence of venous valves located at the thoracic outlet, which prevent the transmission of increased thoracic pressure to the venous circulation. While the thoracic pump theory is the most corroborated in load-distributing-band devices, the cardiac pump theory is more likely to occur in piston-driven devices, even though many factors, such as chest characteristics, ventilation pressure, patient’s age, and device placement, could play a role [6]. Very little is known about the physiology of chest compressions when a combined type, piston, and band is used. From all these considerations, some differences in outcome may be expected when comparing different devices.

Randomized controlled trials [7,8,9,10,11,12], observational studies [13,14,15,16,17,18,19,20,21,22,23,24,25,26,27,28,29,30,31,32], and meta-analyses [33,34,35,36,37,38,39,40,41,42,43,44] have been carried out trying to assess the effect of these devices on the return of a spontaneous circulation (ROSC) and on OHCA patients’ survival, but they led to conflicting results. Moreover, Autopulse^®^ and LUCAS^®^ were the most studied but, once again, very little evidence is available for other devices such as EASY PULSE^®^ [31,32]. A clear and favorable effect on ROSC and survival has never been demonstrated, thus European [45] and American [46] guidelines do not recommend the routine use of these devices, which can be useful in particular settings where high quality CPR may be difficult to achieve.

This study aims to assess whether mechanical CPR affects the probability of ROSC and 30-day survival and if there are differences among devices.

## 2. Materials and Methods

### 2.1. Type of Study and Endpoints

This was a retrospective propensity-score-based analysis of prospectively collected data.

Our primary endpoint was to compare the rate of ROSC in patients treated with mechanical and manual CPR and to seek differences in the rate of ROSC depending on the mechanical CPR device used.

The secondary endpoint was to assess the 30-day mortality according to the CPR type performed. 

### 2.2. Study Population and Data Collection

The study population included all patients enrolled in the Out-of-Hospital Cardiac Arrest registry of the Lombardy region, named “Lombardia CARe”, from January 2015 to December 2022.

Lombardia CARe (ClinicalTrial.gov ID: NCT03197142) is a population-based OHCA registry following Utstein recommendations [47]. Patient enrollments started in the Province of Pavia in 2015. Data collection was subsequently extended to the provinces of Lodi, Cremona, and Mantua in 2019, Varese in 2020, and Brescia and Como in 2021. The Registry was approved by the Ethics Committee of the Fondazione IRCCS Policlinico San Matteo and by all the Ethics Committees of the territories progressively involved. Informed consent was signed only by patients who survived at hospital discharge with a good neurological outcome in agreement with the Ethics Committee.

### 2.3. Territory and EMS Description

The territory covered by LombardiaCARe encompassed an area of 15,126 km^2^, covering a population of 4.243.857 million inhabitants, divided as follows: Pavia 2969 km^2^; 534,506 inh., Lodi 783 km^2^; 227,327 inh., Cremona 1770 km^2^; 351,654 inh., Mantua 2341 km^2^; 404,476 inh., Varese 1198 km^2^; 877,668 inh., Como 1279 km^2^; 594,941 inh., and Brescia 4786 km^2^; 1.253.157 inh. (as of 1 January 2022).

The Emergency Medical Service is provided by the Agenzia Regionale dell’Emergenza Urgenza (AREU), which covers the whole Lombardy region. There are four different EMS dispatch centers, which coordinate rescues among the provinces, named “Sale Operative Regionali dell’Emergenza Urgenza” (SOREU):

- SOREU della Pianura: EMS dispatch center for the provinces of Pavia (PV), Lodi (LO), Cremona (CR), Mantua (MN), and the western part of Milan (MI) province (not yet covered by the Registry); it coordinates 48 ambulances staffed with basic life support and defibrillation (BLS-D)-trained personnel, and 22 advanced life support (ALS)-trained staffed vehicles (a physician and a specialized nurse or a specialized nurse only).
- SOREU dei Laghi: EMS dispatch center for the provinces of Como (CO), Lecco (LC), Monza (MB) (not yet covered by Lombardia CARe), and Varese (VA); it coordinates 56 ambulances staffed with basic life support and defibrillation (BLS-D)-trained personnel and 21 advanced life support (ALS)-trained staffed vehicles (a physician and a specialized nurse or a specialized nurse only).
- SOREU delle Alpi: EMS dispatch center for the provinces of Brescia (BS), Bergamo (BG), and Sondrio (SO) (BG and SOare not yet covered by Lombardia CARe); it coordinates 91 ambulances staffed with basic life support and defibrillation (BLS-D)-trained personnel and 41 advanced life support (ALS)-trained staffed vehicles (a physician and a specialized nurse or a specialized nurse only).
- SOREU Metropolitana: EMS dispatch center for the city of Milan and the eastern part of its province (not involved in the present study).

The specialized nurse, if alone in the ALS staffed vehicle, applies the same ALS protocol, using supraglottic devices (instead of tracheal intubation) and mechanical CPR devices. The decisions about the attempt of resuscitation and its duration are left to the physician. BLS-D-trained personnel are instructed to start resuscitation unless clear signs of death are present (e.g., rigor mortis, hypostasis, and injuries not compatible with life).

All the ALS-trained staffed vehicles were equipped with one of these three different types of mechanical chest compressor: Autopulse^®^ (ZOLL Medical, MA) in the provinces of Lodi and Pavia; LUCAS^®^ (Stryker, MI) in the provinces of Como and Brescia (in the province of Brescia on physician staffed vehicle only); and Easy Pulse^®^ (Schiller, Switzerland) in the provinces of Cremona, Mantua, Varese, and in the province of Brescia in nurse-staffed vehicles only. The decision of using mechanical chest compressors was left to the physician or the nurse in the field (in the case of a specialized, nurse-staffed ALS vehicle).

### 2.4. Data Management and Statistical Analysis

Data were collected and managed using the “REDCap” platform, an internationally used electronic data capture tool, hosted at Fondazione IRCCS Policlinico San Matteo [48].

Statistical analyses were performed with MedCalc^®^ Statistical Software version 22.002 (MedCalc Software Ltd., Ostend, Belgium). Categorical variables were presented as numbers and percentages and compared with Chi-squared test or Fisher exact test; continuous variables were tested for normal distribution with the D’Agostino-Pearson test. If normally distributed, continuous variables were presented as mean and standard deviation (SD), otherwise as median and 25–75 interquartile range (IQR). Differences among continuous variables were investigated by a suitable parametric test (*t*-test or ANOVA) or non-parametric test (Mann–Whitney or Kruskall–Wallis).

By using a multivariable logistic regression model, we tested the association between all the variables supposed to be able to influence the decision of using a mechanical device for chest compressions. From the resulting coefficients, a propensity score was created and tested, according to which two groups of treatment (mechanical and manual CPR) were randomly matched. These two groups were then used for the analysis concerning the primary and the secondary endpoints.

For the primary endpoint, we ran a logistic regression model, both raw and adjusted, testing the association between the use of each one of the devices and the probability to achieve ROSC, assuming manual CPR as a reference.

For the secondary outcome, a Cox regression model, raw and adjusted, was ran, testing the association between the use of each one of the devices and the probability of death at 30 days, considering manual CPR as a reference.

A *p* value < 0.05 was considered statistically significant, with a correct adjustment in case of post hoc multiple comparisons.

## 3. Results

### 3.1. Study Population

**Figure 1:**
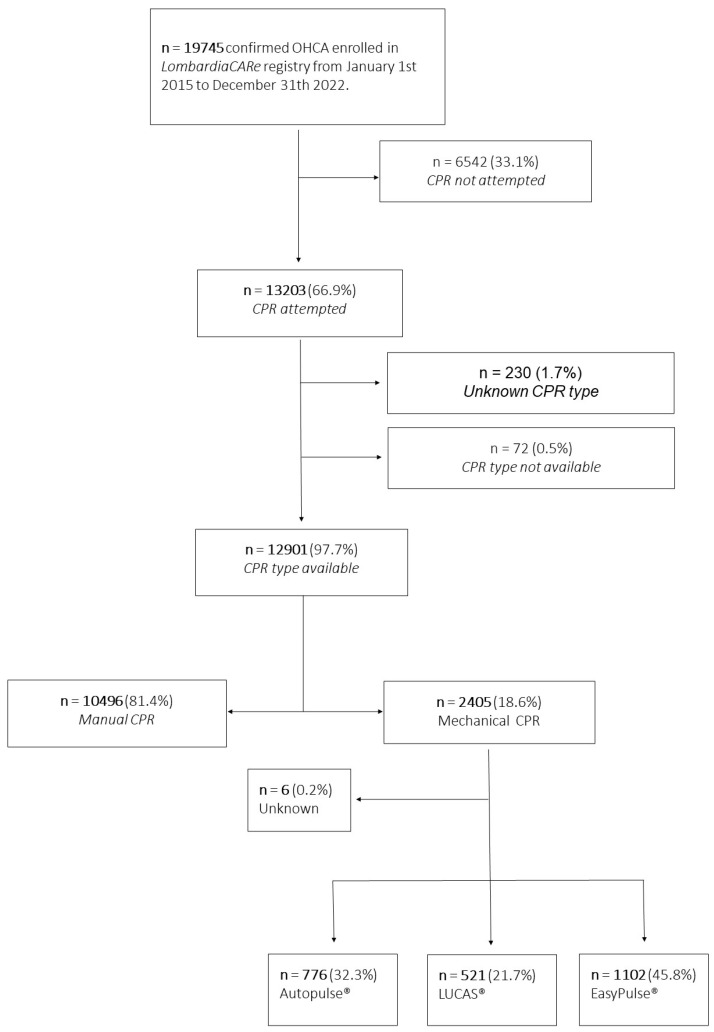
Study population.

Between January 2015 and December 2022, 19,745 OHCAs occurred in the study area, prospectively collected in the Lombardia CARe registry. Resuscitation was attempted in 13,203 cases (66.9%,) and the type of resuscitation was known in 12,901 cases (98%) (mechanical CPR for 2405 (18.6%), and manual CPR for 10,496 (81.4%)) (Figure 1).

Baseline characteristics of the study population, according to the type of CPR received, are summarized in Table 1.

In the group of patients treated with mechanical CPR the percentage of males, of witnessed cardiac arrest at home, of shockable presenting rhythm and of bystander CPR were significantly higher compared to manual CPR; patients were younger and received a more prolonged resuscitation (Table 1).

### 3.2. Primary Endpoint: Use of Mechanical CPR and ROSC

We identified two randomly matched propensity-score-based groups of patients (manual CPR and mechanical CPR) with an identical propensity score (manual: 0.26 IQR (0.16–0.39) vs. mechanical: 0.26 IQR (0.16–0.39); *p* = 1) consisting of 2146 patients each, and the type of device was available in 2142 patients of the mechanical group. Table 2 shows the coefficients for propensity score calculation.

The two resulting groups were homogeneous for the main variables (Table 3). When the group of mechanical CPR was split and the characteristics of patients treated with different devices were compared, we found that they differed for age, gender, EMS arrival time, OHCA location, witnessed status, rate of shockable-presenting rhythm, and resuscitation duration (Table 3).

**Figure 2:**
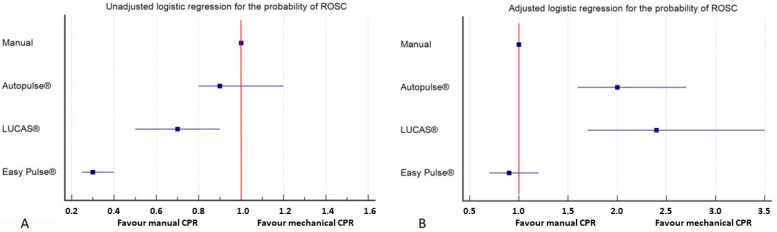
Logistic regression for the probability of ROSC, considering manual CPR as a reference: both raw (panel (**A**)) and adjusted for confounders (panel (**B**)).

ROSC was achieved in 484 (23%) patients in the manual CPR group and in 313 (15%) patients in the mechanical CPR group (χ^2^ = 45.15; *p* < 0.0001). Considering the three devices, the ROSC rates were 156 (22%), 69 (16%), and 88 (9%) for Autopulse^®^, LUCAS^®^, and Easy Pulse^®^, respectively (χ^2^ = 57.6; *p* < 0.0001). The raw logistic regression showed a neutral effect of Autopulse^®^ [OR 0.9 95%CI (0.8–1.29, *p* = 0.66] and a significant negative association with the probability of ROSC for LUCAS^®^ [OR 07 95%CI (0.5–0.9), *p* = 0.004] and Easy Pulse^®^ [OR 0.3 95%CI (0.2–0.4), *p* < 0.001] (Figure 2A). Multivariable analysis, adjusted for age, gender, EMS arrival time, OHCA location, witnessed status, rate of shockable-presenting rhythm, and resuscitation duration showed that the uses of Autopulse^®^ [OR 2.1, 95%CI (1.6–2.8), *p* < 0.001] and LUCAS^®^ [OR 2.5, 95%CI (1.7–3.6), *p* < 0.001] were significantly associated with the probability of achieving ROSC compared to manual CPR, and, on the contrary, the use of Easy Pulse^®^ was not [OR 0.9, 95%CI (0.7–1.2), *p* = 0.66] (Figure 2B).

### 3.3. Secondary Endpoint: Use of Mechanical CPR and 30-Day Survival

**Figure 3:**
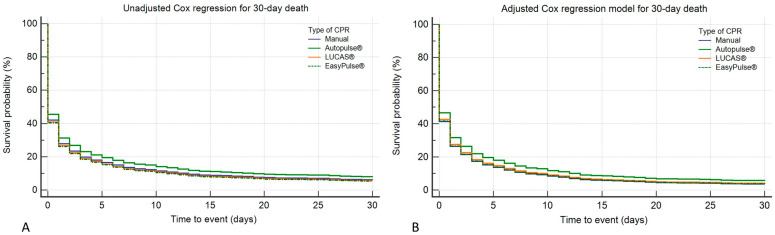
Cox regression models for the risk of death at 30 days, considering manual CPR as a reference: both raw (panel (**A**)) and adjusted for confounders (panel (**B**)).

By comparing the two propensity-score-matched groups, the rate of 30-day survival was significantly lower in patients who received mechanical CPR versus manual CPR [6% vs. 14%; χ^2^ = 80.5; *p* < 0.0001]. Considering the three devices, the rates of 30-day survival were 9.4%, 5%, and 3.4% for Autopulse^®^, LUCAS^®^, and EasyPulse^®^, respectively (χ^2^ = 28.1; *p* < 0.0001). Raw Cox regression analysis showed that only Autopulse^®^ was significantly associated with a lower risk of 30-day mortality [HR 0.9, 95%CI (0.8–0.9), *p* = 0.035] compared to manual CPR (Figure 3A). After correcting for age, gender, EMS arrival time, OHCA location, witnessed status, rate of shockable-presenting rhythm, and resuscitation duration, Autopulse^®^ was confirmed to be significantly associated with a reduced risk of 30-day mortality [HR 0.9, 95%CI (0.8–0.9), *p* = 0.005], whereas LUCAS^®^ [HR 0.97, 95%CI (0.8–1.1), *p* = 0.6] and Easy Pulse^®^ [HR 1, 95%CI (0.9–1.1), *p* = 0.9] were not (Figure 3B).

## 4. Discussion

Our study, one of the few studies comparing not only manual versus mechanical CPR but also the different types of chest compression devices, highlighted that the use of such devices could increase the rate of ROSC, especially in case of prolonged resuscitation. Our study also outlined that the different devices were not equal, and this may have significantly influenced patient outcomes. In particular, the load-distributing-band device was only able to increase the 30-day survival after propensity score matching. As outlined before, this study, different from the majority of previous studies on this topic, provided a comparison of three different devices, exploring their impact both on ROSC and on 30-day survival. This was a valuable point because there was only one paper [13] that considered three devices; however, no direct comparison was provided. Moreover, in that paper, two piston-driven machines and one load-distributing-band device were considered, whereas we compared a load-distributing-band device, a piston-driven device, and a combined band and piston device. In our population, after propensity score matching, the rates of ROSC were higher in patients receiving manual chest compressions compared to those treated with mechanical devices. One randomized trial with Autopulse^®^ [9] and one observational study with LUCAS^®^ [24] found worse outcomes in their mechanical CPR groups. Specifically, our data showed through testing the three devices separately that Autopulse^®^ was similar to manual CPR, and LUCAS^®^ and EasyPulse^®^ were associated with a lower probability of ROSC. However, after correction for confounders, Autopulse^®^ and LUCAS^®^ were associated with a higher probability of ROSC. This suggested the importance of confounders, among which the duration of resuscitation was probably the most important (highest values of Wald test), probably meaning that their impacts on outcomes increase over time. This hypothesis may justify the lack of effect in the aforementioned studies, in which resuscitation duration was not taken into account, and the results of our previous study [25], where the use of a load-distributing-band device increased the rate of ROSC, event survival, and survival to hospital discharge in patients with non-shockable-presenting rhythm, who were more likely to receive prolonged resuscitation. Different resuscitation durations might be at the basis of the existing differences among previous studies because they could have enrolled patients requiring resuscitations of different durations. In two studies reaching neutral results [16,28], resuscitation duration was about ten minutes shorter than in our study, and, in the study by Seewald and colleagues [14], it was found that the longer the resuscitation the clearer the favorable effect.

A positive effect on ROSC was found in one [8] out of six randomized trials and in six [14,15,17,25,29,30] out of eighteen observational studies, whereas meta-analyses showed neutral results [33,34,35,36,37,38,39,40,41,42,43]. The reason why the beneficial effect on ROSC was more evident in observational studies than in randomized ones may be explained by the fact that mechanical chest compressors were compared to very-high-quality manual CPR performances in the randomized trials. The rescuers taking part in the randomized trials were indeed trained, often verified within a short period of time, and re-trained, per protocol, to be sure they retained their high-quality skills. Conversely, observational studies are closer to real life conditions, where rescuers are evidently trained but not so closely monitored to verify their CPR quality; thus, in this setting, it is not surprising that mechanical CPR is more effective than manual resuscitation. Moreover, an element worth discussing is that all the variables included in our model for propensity score calculations were those on which the UB-ROSC [49] score was based. The UB-ROSC score (available at http://www.sanmatteo.org/site/home/ub-rosc-score.html) is an Utstein-based score that is able to predict ROSC in OHCA patients with an area under the curve of 0.8. From here, it shows that the two populations that resulted from propensity-score-based matching had a homogeneous UB-ROSC score and, consequently, a similar a priori probability of ROSC. This was not a negligible detail because this meant that the difference in the rate of ROSC that we found was driven mostly by resuscitation technique.

As far as direct comparison between different devices is concerned, our paper stands out because only limited data are available in the literature. Kim and colleagues [23] ran a propensity-score-based comparison between Autopulse^®^ and LUCAS^®^, and they did not find any differences concerning the rate of ROSC and survival to hospital discharge. We found that these two devices were equally associated with an increased probability of ROSC, but only Autopulse^®^ was also associated with an increased chance of 30-day survival. However, we added a third device in the comparison, and we saw that EasyPulse^®^ was similar to manual CPR without improving neither ROSC nor 30-day survival after correction for confounders.

From a pathophysiological point of view, it is not surprising that the three machines performed differently. According to the classical physiology of chest compressions [6], load-distributing-band devices have different effects on a chest during compressions. Autopulse^®^ was designed according to the thoracic pump theory, with a more predictable mechanism of function. It was shown to be able to improve both cerebral perfusion [50] and coronary perfusion [51] when compared to standard CPR, explaining its positive role on ROSC and survival.

On the contrary, piston-driven devices can act differently according to a patient’s characteristics and the piston’s position on the chest. By using transesophageal echocardiography, LUCAS^®^ was shown to be able to provide more effective chest compressions than manual CPR [52], and this could explain the higher rate of ROSC. However, this effect was shown to be strictly position-dependent [53], and this could explain the better performance of Autopulse^®^, which not only increased the rate of ROSC but was also significantly associated with an increase in survival.

Concerning Easy Pulse^®^, it has a hybrid way of performing CPR, called “circulatory thoracic compression” by some authors [31]. There are two studies [31,32] including this device, and both of them described a lower compression depth compared to piston-driven machines. These two studies found a compression depth of about 35 mm, which was lower than the compression depth obtained with LUCAS^®^ and lower than the 50–60 mm recommended by the guidelines. Even if we do not have data about CPR quality, it is plausible that such shallow chest compressions may negatively affect the rate of ROSC.

Our results may have some practical implications both for rescuers and for manufacturers, as they may serve as a guide to choose the best device and to encourage companies to improve the performances of their products.

### Study Limitations

This study had some potential limitations. The first was that it was not a randomized interventional study. However, the propensity score random matching technique should have mitigated this limitation. The second limitation, consequently to the first, was that the decision of using mechanical CPR was arbitrarily left to the physician on scene, or to the specialized nurse if alone, without a specific standard of procedure protocol upstream. We think that the propensity score random matching technique should have also solved this limitation, at least largely. Furthermore, all emergency vehicles were equipped with only one type of the three machines examined, and we attributed the type of device according to the first ALS vehicle that arrived on the OHCA setting. As, in some cases, more than one ALS vehicle was alerted, it was possible that two ALS vehicles equipped with two different mechanical compressors arrived on the scene. It could not be excluded that the mechanical chest compressor was changed during resuscitation in some rare cases. Even if we were not able to quantify this occurrence, we were rather sure that it was not common enough to bias our findings. A further limitation was that we did not have the exact duration of manual chest compression before mechanical CPR started; reasonably, it was supposed to be similar in the two groups, but we were not able to verify this. The last limitation is that we were not able to provide information about airway management and ventilation strategies, as the Utstein template defined this information as optional. The decision on how to manage airways and the decision of oxygenation targets were left to the physicians or to the specialized nurses in the field, who both followed international guidelines. Recent data showed how ventilation and oxygenation could affect the outcome [54,55,56], but we think that the great majority of patients were intubated and ventilated either manually or mechanically.

## 5. Conclusions

Mechanical chest compressions could increase the rate of ROSC, especially in cases of prolonged resuscitation. Devices for mechanical CPR are not similar, and their different performances could significantly affect patient outcomes. A load-distributing-band device was the only device able to favorably affect 30-day survival.

## Figures and Tables

**Table 1:** Patients’ baseline characteristics.

Variable	Manual CPR	Mechanical CPR	General Population	*p*-Value
	*n* = 10,496	*n* = 2405	*n* = 12,901	
**Male gender, *n* (%)**	5960 (56.8%)	1745 (72.6%)	7705 (59.7%)	<0.001
**Age, years (IQR)**	80.0 (69.0–87.0)	66.0 (55.0–76.0)	78.0 (65.0–86.0)	<0.001
**EMS arrival time, min (IQR)**	13.0 (10.0–16.0)	12.0 (10.0–15.6)	12.8 (10.0–16.0)	0.002
**Medical etiology, *n* (%)**	9735 (92.7%)	2213 (92.0%)	11,948 (92.6%)	0.22
**Home Location, *n* (%)**	8792 (83.8%)	1809 (75.2%)	10,601 (82.2%)	<0.001
**Witnessed status, *n* (%)**				<0.001
Yes—bystander	5095 (48.5%)	1498 (62.3%)	6593 (51.1%)	
No	3750 (35.7%)	604 (25.1%)	4354 (33.7%)	
Yes—EMS	1371 (13.1%)	268 (11.1%)	1639 (12.7%)	
Unknown	280 (2.7%)	35 (1.5%)	315 (2.4%)	
Bystander CPR, *n* (%) *	3413 (37.4%)	1283 (60.0%)	4696 (41.7%)	<0.001
**Shockable-presenting rhythm, *n* (%)**	1176 (11.2%)	724 (30.1%)	1900 (14.7%)	<0.001
PAD Shock, *n* (%) **	76 (25.3%)	42 (40.4%)	123 (29.5%)	<0.001
**Resuscitation duration, min (IQR)**	25.0 (15.0–37.2)	45.0 (32.1–61.5)	28.0 (16.7–42.0)	<0.001

* EMS witnessed excluded, ** PAD applied.

**Table 2:** Coefficients for propensity score calculation and propensity score performance.

Variable	Multivariable Logistic Regression Analysis for Mechanical Chest Compressor Use	
	Coefficient	*p*-Value
**Male gender**	0.35	<0.0001
**Province**		
Brescia	0	ref
Como	−0.28	00036
Cremona	−1.33	<0.0001
Lodi	−1.77	<0.0001
Mantova	−0.54	<0.0001
Pavia	−0.93	<0.0001
Varese	−1.31	<0.0001
**Age (years)**	−0.04	<0.0001
**EMS arrival time (min)**	−0.01	0.024
**Home Location**	−0.19	0.003
**Witnessed event and bystanders CPR (BCPR)**		
No Witnessed/No BCPR	0	ref
No Witnessed/Yes BCPR	0.44	<0.0001
EMS witnessed	0.38	0.0001
Bystander witnessed/No BCPR	0.47	<0.0001
Bystander witnessed/Yes BCPR	0.94	<0.0001
**Shockable-presenting rhythm**	0.75	<0.0001
**PAD Shock**	−0.54	0.04
**Medical Etiology**	0.85	<0.001
**Propensity score performance**	AUC = 0.79; 95%CI (0.78–0.79), *p* < 0.0001	

**Table 3:** Patients’ characteristics according to the type of CPR considering the propensity-score-matched population.

Variable	Manual CPR	Mechanical CPR	*p*-Value	Autopulse^®^	LUCAS^®^	EasyPulse^®^	*p*-Value
	*n* = 2146	*n* = 2146		*n* = 716	*n* = 425	*n* = 1001	
**Male gender, *n* (%)**	1584 (74)	1533 (71)	0.08	560 (78)	305 (72)	665 (66)	<0.001
**Age, years (IQR)**	69 (56–79)	67 (57–77)	0.04	64 (54–72)	69 (58–78)	70 (59–80)	<0.001
**EMS arrival time, min (IQR)**	12 (9.6–15)	12.2 (10–16)	0.17	11.7 (8.4–15)	12 (10–15)	13 (10–16)	<0.001
**Medical etiology, *n* (%)**	1962 (91.4)	1981 (92.3)	0.29	665 (93)	382 (90)	930 (93)	0.11
**Home Location, *n* (%)**	1661 (77.4)	1644 (76.6)	0.54	528 (74)	320 (75)	793 (79)	0.02
**Witnessed status, *n* (%)**			0.26				<0.001
Yes—bystander	1325 (62)	1322 (61)		459 (64)	250 (59)	612 (61)	
No	537 (25)	570 (27)		140 (20)	135 (32)	293 (29)	
Yes—EMS	284 (13)	254 (12)		117 (16)	40 (9)	96 (10)	
Bystander CPR, *n* (%) *	1087 (58)	1107 (58)	0.93	364 (61)	209 (54)	531 (59)	0.53
**Shockable-presenting rhythm, *n* (%)**	566 (26)	585 (27)	0.51	236 (39)	91 (24)	213 (24)	<0.001
PAD Shock, *n* (%) **	32 (33)	34 (40)	0.33	14 (56)	5 (28)	15 (36)	0.14

* EMS witnessed excluded, ** PAD applied.

## Data Availability

Data will be provided upon request to the corresponding author.
